# Excerpta

**Published:** 1881-05

**Authors:** 


### EXCERPTA.

### SULPHATE OF COPPER IN CROUP.

Thibon (*La Lancette Belge*,) says that the effect of sulphate of copper, which he claims to be undoubtedly beneficial, is due to its action as an emetic and a parasiticide. The parasiticide theory is unquestionably a far-fetched one, still the remedy may act as favorably as claimed without necessarily implying the truth of the theory of its action. He gives a half tablespoonful of a mixture of seven grains of sulphate of copper to two ounces of water every ten minutes until vomiting occurs. This is said to be the most rational method of treating croup, and the article claims that complete success will occur from its use, however grave the case.

### MEDICAL EDUCATION.

It seems to us that medical education has reached a crisis in this country which must arrest the attention of the profession and the general public in a serious way. The nefarious transactions of Buchanan, the diploma seller, are too well known to rehearse; but as bad as they are, they are not worse than other phases of the diploma transactions in other parts of the country. In North Carolina a college was chartered by that most notorious revolutionary Legislature of 1868, under the title of the "Edinborough Medical College." This miserable cheat sent an unknown number of men abroad with diplomas, chiefly into South Carolina, and its career of infamy was only ended by the death of *the* only "professor." So far, only one of the graduates of this "college" has presented himself before the Board of Examiners for license, and he was rejected—not because he presented the diploma of a disreputable school, but simply his qualifications, the only test acknowledged by the law of the State.

In looking over the very long list of persons who bought diplomas from Buchanan, as published in the *Philadelphia Record*, we discovered eight belonging to North Carolina. It may be shown that there are more to be added when complete investigations are made, although we hope not. Among this number we recognize two who are now probably practising in this State.

As more and more light is shed upon the uncertainties which surround the issue of diplomas, the public that heretofore amiably considered a diploma from one medical college as the equal of that from any other, and that medical graduation meant the preparation of a student up to a certain general uniform standard, is now having its faith seriously shaken. It is at this crisis in the condition of medical education, that the wisdom of the North Carolina law is so apparent. Every year thinking people are beginning to recognize the necessity of separating the teaching and the examining bodies.

That our people may know something of the extent and character of the work of the Board of Examiners, we append the report of Dr. H. T. Bahnson, Secretary.

### Partial Report of the Proceedings of the Board of Medical Examiners of the State of North Carolina.

---

During the past two years there have been seventy-five applications made to the Board for license to practice medicine in the State. Of these, after personal examination of the applicants, sixty-three were granted, and twelve were refused. With the exception of four, the applicants were graduates of various medical institutions, as follows :

| NAMES OF COLLEGES. | LICENSED. | REJECTED. | TOTAL. |
|---|---|---|---|
| Jefferson Medical College, Philadelphia | 5 | ...... | 5 |
| *Washington University, Baltimore* | *13* | *3* | *16* |
| *University of Maryland* | *8* | ...... | *8* |
| Long Island Hospital College | 1 | ...... | 1 |
| Bellevue Hospital Medical College | 6 | ...... | 6 |
| Louisville Medical College | 5 | ...... | 5 |
| Kentucky School of Medicine | 1 | 1 | 2 |
| Philadelphia University | 1 | ...... | 1 |
| University of New York | 10 | ...... | 10 |
| University of Pennsylvania | 2 | ...... | 2 |
| Medical College of South Carolina | 3 | 1 | 4 |
| *College of Physicians and Surgeons, Baltimore* | *3* | *2* | *5* |
| *Baltimore Medical College* | *1* | *1* | *2* |
| University of Virginia | 1 | ...... | 1 |
| Central University of Louisville, Kentucky | ...... | 1 | 1 |
| McLean's School (North Carolina) | ...... | 1 | 1 |
| Unknown | ...... | 1 | 1 |
| *Non-graduates* | *3* | *1* | *4* |

The year of graduation of rejected applicants, was as follows:

Washington University, two in 1868, and one in 1872.

Kentucky School of Medicine, one in 1876.

College of Physicians and Surgeons, Baltimore, one in 1872 and one in 1879.

Charleston Medical College, one in 1876.

Baltimore Medical College, one in 1876.

Central University of Louisville, Kentucky, one in 1875.

McLean's School (N. C.), one in 1875.

HENRY T. BAHNSON, M. D.,

Secretary Board of Med. Examiners, of N. C.

Salem, N. C., February 9th, 1881.

It will be seen that out of *seventy-five applicants*, *twelve were rejected*, and even at this rate, the standard of examinations was by no means as high as the Board wished to make it. It was painfully evident to the most earnest friends of the Board, that if a higher standard had been maintained, twice as many would have failed. If the people of the State were fully aware of the experiences which have been accumulated by the Board, they would demand a more stringent law than the one now in force.

NOTE.—The foregoing article on "Medical Education," is taken from the *North Carolina Medical Journal*, and cannot fail to interest all who may peruse it. Being official, its statements are entitled to implicit credence. It brings to light in a most painfully convincing manner, the *little value* certain medical diplomas are, as evidence of the fitness of the holders of them to discharge the most responsible duties of physicians. We have placed the names of our Baltimore Colleges and their graduates, in italics, so that our home readers may see the status of some of those receiving diplomas in this city, who were before the State Medical Board of North Carolina. We have also italicised the line of "non-graduates," to show that they who had not graduated at any medical college whatsoever, were successful in a larger per centage than were the graduates of one of our colleges! We know nothing of the Baltimore Medical College which two of the applicants named as their alma mater, but by reference to the last paragraph quoted, it will be seen, that had the examination been more strict, the showing from one of the schools might have been more pitiable still. EDS. IND. PRAC.

### THE MANAYUNK ODONTALGIACAL COLLEGE AND HOSPITAL OF UMBILICAL SURGERY.

*Spring Announcement.*—In order to increase the facilities for obtaining diplomas, the Faculty of this College assumes to matriculate students by mail, telegraph or telephone.

*Mail.*—Arrangements have been perfected through which the ridiculous formality of attendance upon the courses of instruction may be entirely dispensed with. The Faculty assumes to direct the reading of the matriculate from time of birth. Names may be entered by mail.

*Telegraph.*—Telegraphic communications will be established with the home of each matriculate in any part of the world. Attendance upon lectures may be conducted by telegraph. Highly interesting, important, amusing, and diversified clinical lectures on umbilical surgery and allied anal diseases will be furnished by telegraph to all parts of the world The anatomico-physiologico-histologico-pathological expressions of each disease will be lucidly formulated, and the harmonistic co-relation completely established in the umbilical mansion, which is thus constructed, and which will give safe refuge to the practitioner.

*Phonograph.*—Theses may be deposited and recorded in the phonograph.

*Telephone.*—Final examinations will begin at the beginning of each session and will be conducted by telephone. The very worthy and highly honorable degree of D. U. S. and D. P. will be conferred by telephone. The candidate is expected to bow to the honorable President of the honorable Board of Trustees and honorable Faculty as he approaches the diaphragm of the telephone and receives the degree. B. Low, D. U. S. & D. P., Dean.

The full force of this can be appreciated only by the initiated·—Ed.

### THE TREATMENT OF MALIGNANT PUSTULE.

At a meeting of the "Societe de Chirurgie" Mr. Boisset alluded to the fact that the treatment of malignant pustule and malignant œdema by intestinal injections of iodine, is at present in great vogue, and expressed his regret at not receiving the credit to which he believes himself entitled in connection therewith. The fact is, that ever since 1855 he has advocated the use of iodine in various forms of anthrax ; he recommends it for its anti-virulent qualities, not only in charbon, but also in farcy ; iodine is far more efficient than the actual cautery, because in the shape of tincture it penetrates within the tissues, and reaches far deeper ; therefore, when injected in the tissues they are so energetically acted upon that a sort of barrier is thrown around the evil, and its further progress is permanently checked. The effects of the iodine are greater in proportion to the concentration of the tincture.

Brownville, Saline County, Mo., February 9th, 1881.

*Dr. A. Flint.*

Dear Sir: Recently I have cured two cases of croup—by croup I mean exudative laryngitis. The following treatment was adopted in both cases. Kerosene or coal oil was given freely in doses of a teaspoonful for four days and nights. At first it was given every hour; afterward every two, three, four and five hours. There certainly is no danger in its free administration. At the same time, fluid ext. jaborandi was given in doses ranging from ten to twenty drops, and repeated just often enough to maintain free respiration. The atmosphere was kept warm and moist, as advised by Dr. Sayre. I believe the coal oil will limit the exudation, and that the jaborandi will produce and maintain a moist and soft condition of the exudation and mucous membrane of the larynx and trachea. Some of the exudation was expectorated a number of times; the presence of it in the expectorated mucus proved conclusively the true character of the disease. I never saw such cases cured before. A very slight mucus rattle was the first favorable symptom in both cases, and as the rattle increased the wheezing, ah! that terrible wheezing, disappeared, and on the fifth day the exudation began to give way. Both the medicines were given six days and nights. I do not think there was any further exudation after six hours' treatment. In both cases the disease was, I believe, of two or three days' standing before treatment commenced. I do hope that these medicines may prove curative in this grave disease. They are certainly worthy of a fair trial. Very respectfully,

D. J. Parsons.

Note.—Dr. Flint has kindly placed this letter before the readers of this Journal.—Ed. *Medical Bi-Weekly.*

### IODOFORM IN CHRONIC NASAL CATARRH.

Dr. H. A. Eberle, of Iowa, writes to the *Michigan Medical News*, of January 10:—

In this State, where catarrh is so prevalent among the people, any remedy that would perform a cure will be hailed with the greatest delight. Iodoform, as a remedy for chronic ulcers, was used quite extensively in the Montreal General Hospital in 1872 to 1876, with such good results as to warrant its trial in private practice. Since that time I have made use of it in very many ways, in the treatment of piles, fissures, granular ulcerations of the uterus, etc., and always with gratifying results.

In view of all its healing properties, I was led to adopt the remedy for the treatment of catarrh, and as I had been a sufferer from the disease in a chronic form for many years, and had used many preparations with varying success and little benefit, I concluded to make use of it in the following manner. First, an ointment is made. thus:

℞. Iodoform, finely powdered, . . grs. lx;
Ext. geranium, solid, . . . . grs. x;
Acidi carbolici, . . . . . gtt. xv;
Cosmolinæ. . . . . . q. s. . . ℥ j.
M.

Secondly, bougies are made out of absorbent cotton saturated with the above ointment and simply introduced up the nasal passage as far as necessary, at bedtime. These are left in all night, and are easily removed in the morning by blowing the nose.

This is repeated for a week or ten days, when the most obstinate case of catarrh will yield to the treatment. Scarcely any other treatment is necessary, except the occasional use of the posterior nasal douche with some cleansing fluid. I usually employ a weak tepid solution of chloride of sodium before introducing the "iodofodized cotton" tent.

### TWO YARDS OF INTESTINE REMOVED.

A Paris letter in an English contemporary describes a remarkable case in the practice of Dr. Kœberle, of Strasburg.

The patient, a young woman, aged twenty-two, had been subject for some years to acute attacks of abdominal pain, and on two occasions there had been symptoms of obstruction, which had, however, been overcome by the use of enemata. Since that time (October, 1880) the pain had been most constant and severe, not remitting day or night, and at times so intense that it could scarcely be soothed by hypodermic injections of morphia. Gastrotomy was performed on November 27th, 1880, and four narrowings were found in the bowels, one being only four millimeters in width. These strictures were distributed over two yards of the gut (2 meters .05), which was excised between two ligatures at each extremity, the vessels of the mesentery being secured by twelve ligatures. The ligatures of the ends of the intestine were then tied together so as to place the gut in the most favorable position for enterotomy, which was performed on the third day. The parts beyond the ligatures came away between the twelfth and fifteenth days, and on the twentieth the first alvine evacuation occurred. Five days later a band of strapping sufficed to prevent food or gas passing through the wound, which had entirely healed in six weeks. The operation was not performed antiseptically, and the temperature never rose beyond 38° Centigrade. During convalescence, and from the third day, the patient was fed by the mouth, with solid and substantial food—bread, meat and eggs—sufficient liquid being given for the purpose of digestion only, the thirst being assuaged by injections of water in the rectum, seventy such injections having been made in the twenty days. The young woman, who is now quite well, has no pain or digestive trouble of any kind.—*Med. and Surg. Reporter.*

### COMPLETE EXTIRPATION OF THE UTERUS, WITH BOTH OVARIES.

Dr. Thomas Chambers reported this case to the London Obstetric Society:

Jane S., aged 45, was admitted into the Chelsea Hospital for Women, May 24th, 1880. In 1870 she first noticed a lump in her right groin, which grew slowly for five years. After this menorrhagia commenced, and gradually increased. Pain and hemorrhage were excessive, and she eventually became too weak to attend as an out patient, and was remarkably emaciated. The tumor was freely movable from side to side, and was very soft and doughy, but without fluctuation. The pelvic cavity was unoccupied, the vagina drawn up into a cone, with the os and cervix, both small, in the centre. There was a periodical discharge of watery fluid through the vagina. Hence a diagnosis of fibro-cystic tumor was made. Medical treatment having proved of no avail, extirpation of the uterus was proposed to the patient, and she decided in favor of the operation. It was performed on June 22d. The abdominal incision was extended to eleven inches. The broad ligaments were tied with silk at each side, a parallel clamp placed above the ligatures, and the uterus cut away. After a few minutes arterial hemorrhage occurred from a large vessel, which was at once secured. The cervix was then transfixed by a double ligature, the clamp removed, the ligatures tightened, and the stump replaced. By the 21st day the patient was convalescent, a free discharge of offensive matter from the vagina having taken place suddenly on the 14th day. The tumor proved to be a lobulated white fibroid, not fibro-cystic, and contained very large vessels.

### ON THE CONTROL OF DIARRHŒA IN TYPHOID FEVER.

Dr. James W. Allan, superintendent of the Glasgow Fever Hospital, says, in the *Lancet*, March 19:—

It is, perhaps, better not to attempt to check the diarrhœa of enteric fever so long as it is mild—that is to say, as long as the motions do not exceed three or four in the twenty-four hours. But when the stools are very loose and copious, as well as frequent, it becomes a very desirable thing to control, if not to stop, the diarrhœa. Severe purging rapidly exhausts a patient. In the case of children the following measures may be tried:—(1) Boiling the milk which constitutes patient's diet; (2) boiling cinnamon in the milk, and straining it; (3) adding lime-water to the milk in varying proportions, say from one in four to half in half.

The above simple remedies are well worthy of trial. They have the great advantage, in the case of children, of not being "bad to take," and further, while the patient is taking the medicine he is taking his diet (*i. e.*, milk) at the same time.

In the case of adults the means just mentioned should first of all have a trial. If they fail we may then try (1) that excellent pill—lead pill with opium—say one every three or four hours, till the diarrhœa is restrained. This pill is very valuable in the treatment of purging, and it has the additional advantage of tending to relieve pain and check flatulent distention of the intestines. Should the purging continue, and especially if the desire to go to stool be urgent and persistent, we should at once resort (2) to the use of the starch and laudanum injection—say ten, fifteen or twenty drops of laudanum in two tablespoonfuls of thick starch, injected into the rectum. This is a capital remedy; it checks the diarrhœa, allays the irritation, and probably at the same time disposes the patient to sleep. It is perhaps necessary to add, that no beaf-tea should be given while there is diarrhœa.

### *AIR AND VENTILATION.

So much has been written of late years about the necessity of ventilation, that we feel that we ought to apologise for calling your attention to facts with which all should be familiar. We would not do so had we not been so frequently struck with the deplorable ignorance, everywhere met with, of the simplest principles of hygiene in connection with atmospheric air, and the consequent violation of its plainest requirements. Contaminations of food and water are more readily recognised, because they offend the taste; but impurities of the air are frequently not appreciated by the senses, and their injurious effects are more gradual and more insidious. *Practically, the community is not alive to the fact that impure air is a poison, and is directly or indirectly the cause of great mortality;* this, too, notwithstanding the fact that the necessity of pure air for the integrity of function of human beings has been recognized for many years.

No educated person denies the importance of ventilation; but scarcely one in a hundred gives enough practical attention to it. Many insinuate that it is a hobby of the doctors. Still greater numbers do not really appreciate what is meant by the word ventilation. "It *means running air, just as a river means running water." Air in motion—continually and unceasingly changing—never remaining stagnant*—air coming in from the outside and going out from the interior to the exterior atmosphere.

Before showing how absolutely necessary ventilation is, let us for a few moments glance at the composition of air, which so far as regards its essential elements is the same in all parts of the globe, and at all elevations within our reach! It is a vast ocean in quantity, extending nearly to 55 miles around this little planet where we reside. Thus nature in her bountifulness has furnished us with an inexhaustible supply!

Air is composed of one-fifth of oxygen, four-fifths nitrogen, with an almost infinitesimal proportion of carbonic acid—$\frac{1}{2500}$ of the

*From the Author's Paper on "House Air," &c, atmosphere—with very slight traces of ammonia; aqueous vapor in the proportion of ½ to 2 per cent. This last fluctuates greatly, and is mainly influenced by temperature; at a given temperature, air cannot contain more than a certain proportion of moisture, for it saturates it; generally the air contains from 50 to 75 per cent. of the amount requisite for complete saturation—the average, according to Prof. Wilson, being 1.46 to 100 parts. If the quantity be not within these limits, the air is either unpleasantly dry or moist. The ammonia does not exceed one part in a million parts of air.

Outside air near the earth frequently contains other aeriform substances, and some of a solid fluid nature, gases generated by combustion and decomposition, traces of nitric and acetic acid, besides pollen of plants, spores and germs of low order of living beings. These, however, are accidental constituents.. The currents of air and the winds, the heat and electricity, keep the atmospheric outdoor air in almost ceaseless motion, and these extraneous matters are swept away. The quantity of oxygen is always sensibly diminished in the air of towns, according to Prof. Wilson. The vegetable kingdom, by its absorption of carbonic acid and giving off of oxygen, contributes in no small degree to the purity of the external air. Moreover, the air contains within itself the means of its purification. By a process of oxidation it converts all organic substances exposed to it into simpler forms of matter, such as carbonic acid, nitric acid, water, &c.

*This air is the chief factor of life, and its use in breathing is the first and the very last act of our existence as independent beings.* We can live a certain period of time without food, but we cannot without air. We must breathe from fifteen to twenty times per minute, and not less than 20,000 in 24 hours.

So essential are these respiratory movements and this air to life, that to be deprived of one or the other for five minutes or less is sufficient to produce death! "Starvation," says Parkes, "is a matter of days without solids, hours without liquids, but of minutes without air."

To understand thoroughly the effects of impurities of the atmosphere, we must glance, although hurriedly, at the physiological effects of pure air. The *oxygen* is the element which is efficient in respiration. It is important that it should be understood that the oxygen we breathe enters not only the lungs, but passes, while in the lungs, through the walls of the blood-vessels, and becomes incorporated with the blood, and permeates every tissue and part of the body, and is absolutely necessary for their physiological functions. *Respiration is not confined to the lungs, but extends throughout the body.* In the tissues oxygen is given off, and carbonic acid, the result of the disintegration, and consequently, an impure and effete gas, formed in the tissues, passes into the blood and colors it dark. In the lungs there is absorption of oxygen and discharge of carbonic acid. The cause of this *internal respiration* is explained by physiologists to result from the differences between the tension of the gases of the blood and the atmosphere, respiration equalizing these tensions. An animal, human or other, placed in a confined space, can consume almost the whole of the oxygen which the air contains, whilst the evolution of carbonic acid is very soon stopped by an equalization of the tensions taking place. *The essence of respiration consists in the interchange of these gases*—the appropriation of oxygen and exhalation of carbonic acid. If this interchange does not take place, life becomes extinct. Any impairment of the process, or any impurity of the air, deranges all the functions of the body, such as those of the stomach, nervous system, the brain, the kidneys, &c. This, then, must never be lost sight of, that the lung process is only a *very small part* of respiration, which includes the whole organism. Without the due amount of oxygen the blood does not circulate well in the minutest blood-vessels, and every function is deranged. *We have thus an internal atmosphere of the tissues, as well as an external one.* We pant for breath because our organs and structures everywhere demand oxygen.

At each inspiration the volume of oxygen absorbed is five per cent. of the volume of atmosphere, and the exhalation of carbonic acid four per cent. In the expired air we not only have this amount of carbonic acid, but we have ammonia, and aqueous vapor loaded with organic matter. This last substance can be easily shown by breathing on a sponge, or by allowing the breath to be condensed in a glass vessel. Putrefaction, a property characteristic of organic substances, will take place. In the open air the expired air with its impurities acends and is scattered far and wide; it is purified, and we do not rebreathe it, for we are in literally an ocean of fresh air. The injurious gases become diffused and decomposed; animal emanations are absorbed in vegetation; suspended substances fall to the ground, and organic substances are oxydised, and thus rendered harmless.

Air is not only essential when taken into the organism, but *atmospheric pressure* over every point of the body knits the animal framework firmly together. When we ascend mountain heights, where the atmosphere is lighter, and consequently the pressure diminishes, we suffer with oppression of breathing, palpitation, and many painful symptoms. Under the normal atmospheric pressure the lungs are filled with air as the walls are expanded. These mechanical effects are essential.

We must also bear in mind the physical influence of the atmospheric air in regulating the *animal temperature.* Radiation of the animal heat is from all points into the external air. If the external air be cooled far below the level of the body, then the radiation is so vigorous that we arrest thereby the vital processes of the body; the grand nutritive acts cannot go on, and we must die. *Thus air is food—the first and the last we partake of.* Its oxygen is carried by those wonderful little missionaries, the red globules, throughout every portion of the body, and we live upon it. Without air, food through the stomach cannot be appropriated. In plain language, inspired air is designed to convey to the lungs their natural food, for they consume a part of it just as much as the stomach does bread. Expired air is designed to convey out of the body the foul or impure rinsings from 900,000,000 air-cells which the lungs contain.—*Prof. Donaldson on Domestic Hygiene.*

### THE ETIOLOGY OF DIPHTHERIA.

Dr. Hubert Airy has made a report to the Local Government Board of England, of the results of his investigations into several local outbreaks of diphtheria.

The disease did not seem to be affected by elevation or dampness of site, nor by foulness nor over-crowding. He found abundant instances of its contagiousness, as in schools, from visits to the dwelling of a diphtheria patient, from making purchases at a shop where the shopwoman had diphtheritic sore throat. There was one striking case of the conveyance of diphtheria to a new locality, by a person who had been in contact with a diphtheritic patient, but had not herself contracted the disease. This would show that the poison can attach itself to the person or clothes of a visitor or occasional attendant, and be thus transported for some distance. Dr. Airy concludes that diphtheria is caused by an organism which can multiply both within and without the human body, its increase in the latter case being promoted by clay soils, by the season and by moisture. It is also capable of infecting both water and milk and seems to flourish best in the autumn months.—*British Med. Journal.*

Early Maternity.—Few cases have been reported of maternity having occurred as early as in the famous case of Carus. Dr. Henry Dodd (*London Lancet*, April 5, 1881) reports a case of a child who began to menstruate when twelve months old, and became pregnant at the age of eight years and ten months. Whether or not this precocity is not an atavistic tendency may be readily asked, as it occurs most frequently among the lower races. Dr. Henry Dodd's case is a well authenticated one, as he delivered the girl's mother of her nine years and five months previous.

Asthma.—Good results are claimed for the following mixture of morphia, strychnia and atropia, in the treatment of the asthmatic paroxysm: Bi-meconate of morphia, one-eighth of a grain; atropia sulphate, one one-hundred and twentieth of a grain, and strychnia, one-sixtieth of a grain, given hypodermically and repeated if necessary.

